# Total energy expenditure in adults aged 65 years and over measured using doubly-labelled water: international data availability and opportunities for data sharing

**DOI:** 10.1186/s12937-018-0348-8

**Published:** 2018-03-26

**Authors:** Judi Porter, Kay Nguo, Simone Gibson, Catherine E. Huggins, Jorja Collins, Nicole J. Kellow, Helen Truby

**Affiliations:** 1 Department of Nutrition, Dietetics & Food, Monash University, Level 1, 264 Ferntree Gully Road, Notting Hill, VIC 3168 Australia; 2 Allied Health Clinical Research Office, Eastern Health, 5 Arnold Street, 3128 Box Hill, VIC Australia

**Keywords:** Total energy expenditure, Energy requirement, Doubly-labelled water, Older adults, Data repository

## Abstract

**Background:**

Increasing population lifespan necessitates a greater understanding of nutritional needs in older adults (65 year and over). A synthesis of total energy expenditure in the older population has not been undertaken and is needed to inform nutritional requirements. We aimed to establish the extent of the international evidence for total energy expenditure (TEE) using doubly-labelled water (DLW) in older adults (65 years and over), report challenges in obtaining primary data, and make recommendations for future data sharing.

**Methods:**

Four databases were searched to identify eligible studies; original research of any study design where participant level TEE was measured using DLW in participants aged ≥65 years. Once studies were identified for inclusion, authors were contacted where data were not publicly available.

**Results:**

Screening was undertaken of 1223 records; the review of 317 full text papers excluded 170 records. Corresponding or first authors of 147 eligible studies were contacted electronically. Participant level data were publicly available or provided by authors for 45 publications (890 participants aged ≥65 years, with 248 aged ≥80 years). Sixty-seven percent of the DLW data in this population were unavailable due to authors unable to be contacted or declining to participate, or data being irretrievable.

**Conclusions:**

The lack of data access limits the value of the original research and its contribution to nutrition science. Openly accessible DLW data available through publications or a new international data repository would facilitate greater integration of current research with previous findings and ensure evidence is available to support the needs of the ageing population.

**Trial registration:**

The protocol was registered with the International Prospective Register of Systematic Reviews (PROSPERO), registration number CRD42016047549.

**Electronic supplementary material:**

The online version of this article (10.1186/s12937-018-0348-8) contains supplementary material, which is available to authorized users.

## Background

The challenges of the global ageing population have been well documented, including the social [1], economic and impacts on the health system [2, 3]. The United Nations forecast highlights the progressive nature of this ageing, “the number of older persons has tripled over the last 50 years; it will more than triple again over the next 50 years” [4]. It is predicted that the proportion of individuals aged over 80 years will increase fourfold to reach 4.1% of the world population in 2050. This milestone age group can be referred to as the ‘older elderly’, and are projected to total almost 379 million by 2050 [5].

The changing demographic of our globally ageing population has implications for both nutrition science and practice application. This practice at the population, community and individual level should be informed by the evidence base to ensure outcomes are optimised and resources are used efficiently. Energy and nutrient requirements are fundamental concepts in nutrition science, however a synthesis of the evidence for total energy expenditure in the older population has not previously been undertaken. A greater understanding of the energy needs of the older population will facilitate evidence translation to inform clinical practice and food provision at individual and broader system levels.

Doubly-labelled water (DLW) is the gold standard technique for measuring total energy expenditure (TEE) in free-living individuals. The first application of the DLW method in humans was published in 1982 by Schoeller and Van Santen [6] but its cost and analytical complexity has meant it remains a research tool rather than a method utilised in clinical practice. The review of Black et al. [7] in 1996 synthesised published and some previously unpublished TEE data assessed using DLW of 574 free-living people from affluent societies within the 2–95 years range. Yet to be undertaken however is an analysis of the evidence in the older population. In the first stage of an analysis of the energy requirements of older adults (65 years and over), we aimed to establish the extent of the international evidence for TEE using DLW, report challenges in obtaining primary data, and make recommendations for future data sharing.

## Methods

Data identification were undertaken using principles of a systematic review, hence we followed and reported against the Preferred Reporting Items for Systematic Reviews and Meta-Analyses (Additional file 1: PRISMA) guidelines [8]. Prior to commencement, the protocol was registered with the International Prospective Register of Systematic Reviews (PROSPERO: http://www.crd.york.ac.uk/PROSPERO), registration number CRD42016047549, specifying the rationale, purpose and methodology for the review. This report focuses on the first component of the registered protocol: identifying and describing the process and availability of TEE data obtained via the DLW method. Analysis of participant level data will be conducted and reported later.

### Inclusion and exclusion criteria

Eligible studies included those with participants aged 65 years and above, and where the population was heterogeneous in age but participant-level data could be obtained for those aged ≥65 years. This age is classified internationally as the age for ceasing paid employment [4].

The intervention or assessment method of interest was DLW. This technique [9] is recognised as the reference method for the measurement of TEE and has been used in many populations, medical diagnoses and age groups. It is non-invasive to participants, and has been recognised as a technology that is restricted due to the high cost of the stable oxygen isotope and the technical complexity associated with analysis [10]. The primary outcome was participant-level TEE. For the purposes of this review, DLW measurements were accepted as reported irrespective of the variability in laboratory techniques (e.g. two-point and multi-point methods, duration). Accuracy of the DLW method has previously been estimated at ±5% [7]. Where TEE was measured longitudinally (e.g. in the case of intervention studies), only the baseline measure was included.

Studies where participant-level data were obtained, including validation/comparison studies (e.g. comparing DLW with accelerometer), cross sectional or interventional studies were eligible for inclusion. Reviews, conference proceedings, and editorials were excluded. See Table 1 for summary of inclusion criteria.

**Table 1:** Inclusion criteria for the review of DLW studies in adults aged 65 years and above

Parameter	Description
Population Intervention/exposure Comparison Outcomes Study design	Adults aged 65 and over Doubly-labelled water No comparator Total energy expenditure Full text papers of any study design where participant-level data were available

### Search terms and strategy

**Fig. 1:**
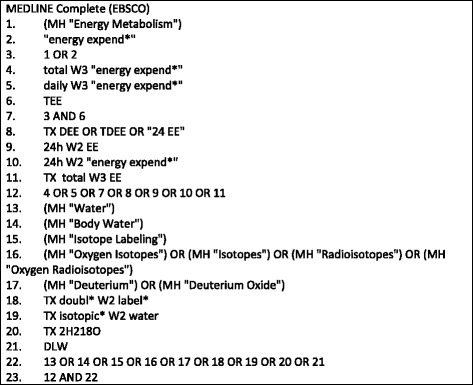
Search Strategy for MEDLINE complete

The search strategy used a variety of subject headings and synonyms relevant to the research question and was developed with an experienced systematic review search librarian. Search terms focused on the intervention and outcome, with age not considered within the search strategy so as not to unnecessarily limit the final library. The literature search was undertaken in MEDLINE complete, EMBASE, CINAHL Plus, and Cochrane Central from database inception to July 2016, with no language or date restrictions applied. The search strategy for MEDLINE complete is described in Fig. 1**,** with similar approaches applied in other databases. The reference lists of relevant systematic reviews were also searched to identify any papers that may have been missed by the search strategy.

### Study selection

Searches were exported to Endnote with duplicates manually removed. Three contributors screened titles and abstracts independently, and in duplicate, to identify studies meeting the inclusion criteria. Any disagreement was resolved by consensus. Full text review was then undertaken by two authors in duplicate to confirm eligibility of studies.

### Data collection and extraction

Corresponding authors were contacted by email by a member of the review team. One follow-up email was sent approximately one month following the initial approach. The purpose of the correspondence was to determine whether their study met the age criteria, and whether they had access to the original data to contribute to this review. An exemption from ethics approval was obtained from the University Human Research Ethics Committee (Project number 8025) for contacting authors and for requesting the collection of these data. Studies where participant-level data were available (either from data extracted directly from published papers inclusion or provided by authors) formed the final library for this review.

Other data sought from authors (or obtained, where reported, in published papers), included resting metabolic rate, age, weight, height, reported/known illnesses, and ethnicity.

The nature of authorship, with different first or corresponding authors within research collaborations and moving institutions led to some groups being contacted on more than one occasion for data. Data from the same original data set were also analysed and presented in multiple publications on several occasions, further confounding the data search. Duplicate data were minimised by grouping studies from the same data set together, as well as referring to the methods section of the papers to identify potential duplicate reporting of TEE data.

A spreadsheet was developed for management of all eligible papers and included identification of the publication and authors, whether any author could be contacted electronically, whether a response was obtained (regarding age of participants, data availability and contribution of data to the review). A second data extraction spreadsheet listed data provided by individual authors/research teams and, data extracted directly from published papers, where reported. Accuracy of data entered into spreadsheets was independently reviewed by two authors.

### Quality assessment

Quality assessment of individual studies will be reported with the publication of primary data.

### Method of analysis

The analysis presented in this paper is a descriptive synthesis of data availability, and participant-level data that has been obtained for further analysis. No primary analysis nor meta-analysis has been included here but will be reported later.

## Results

**Fig. 2:**
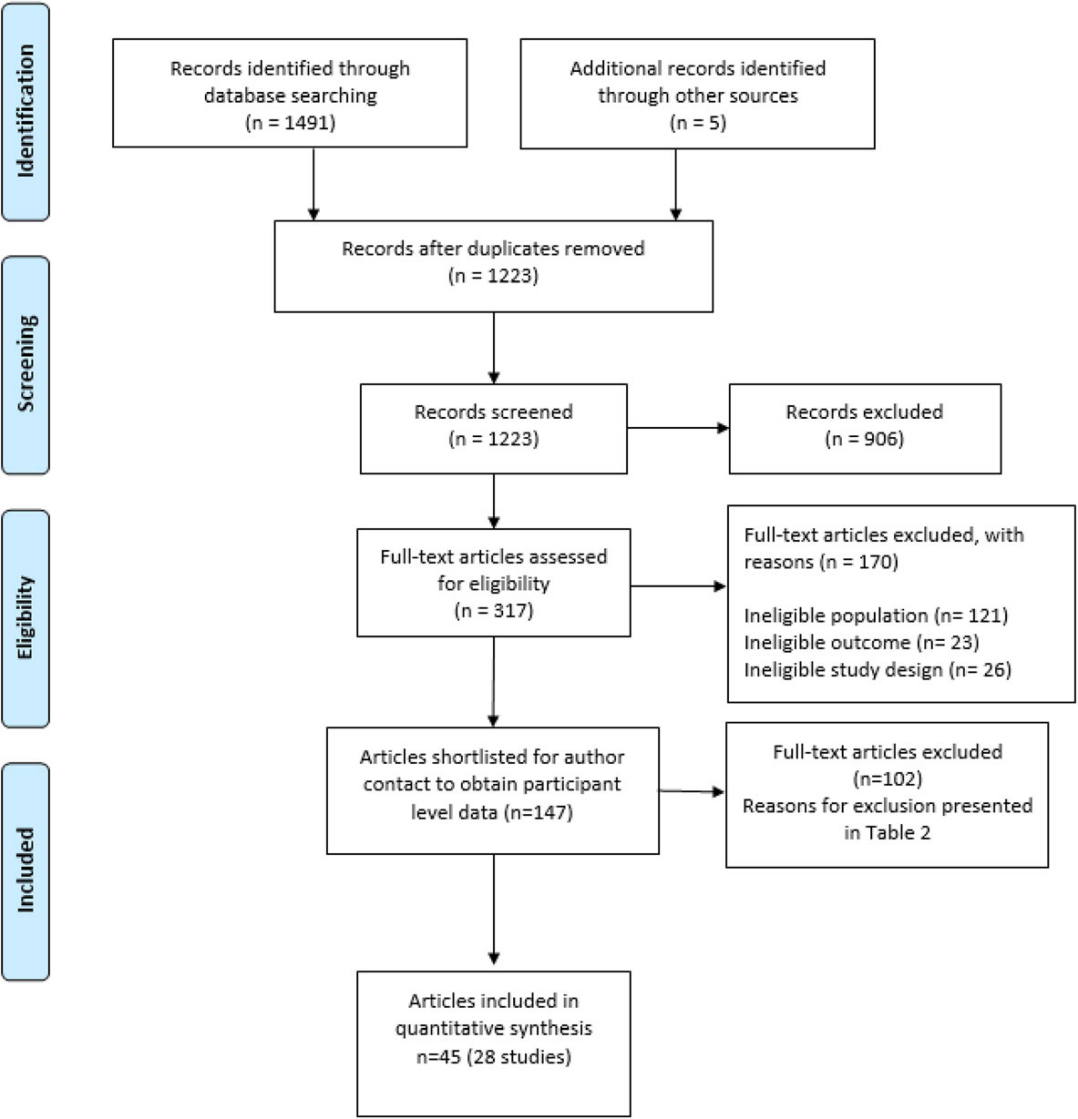
PRISMA flow diagram

Database searching yielded 1491 records, with a further five studies identified through other sources as indicated in Fig. 2**.** Following the removal of duplicates, 1223 records were screened. The review of 317 full text papers was undertaken that excluded a further 170 studies. Most papers excluded at this full text review were due to the wrong populations (e.g. age < 65 years), the wrong outcome (e.g. energy expenditure not measured using DLW), or wrong study design (e.g. conference abstracts).

Authors of the remaining 147 studies were contacted by email to request provision of participant-level data for TEE and other variables. A summary of the outcomes of these communications is included in Table 2**.** Ten papers were able to be excluded as authors confirmed that study participants were all aged younger than 65 years, one additional paper included repeat data collection within a longitudinal study [11]. Three corresponding authors declined to participate, with 11 publications identified from these research groups. Therefore, a total of 102 papers of the 147 shortlisted papers were unavailable or excluded due to ineligible age/duplicate data.

**Table 2:** DLW data availability in the systematic review of DLW in adults 65 years and over

Total number of publications shortlisted for author contact to obtain participant level data (*n* = 147)	
Data availability	Number of eligible publications (*n*=136^a^; %)
DATA THAT ARE UNAVAILABLE	
Data no longer retrievable by authors	34 (25.0)
Corresponding or primary author emailed twice with no response^b^	43 (31.6)
Authors declined to participate	11 (3 groups of authors)(8.1)
Data available: have not yet been provided by authors	3 (1 group of authors) (2.2)
*Total publications where data are unavailable*	***91 (66.9)***
DATA THAT ARE AVAILABLE	
Data available: participant-level data extracted directly from published paper	15 (11.0)
Data available: participant-level data provided by authors	30 (22.1)
*Individual publications where data are available*	***45 (33.1)***
EXCLUDED DATA (ineligible)	
Total publications where no participants were 65 years or over	10
Publications where data were recorded longitudinally	1
*Total excluded data*	***11***

^a^147 studies minus the 11 ineligible studies

^b^Where no contact was made with authors, it was assumed for calculating data availability that these studies met the age criteria for inclusion

Forty-five publications (28 studies) reporting on TEE in individuals aged ≥65 years thus remain in the data set **(**Table 3**).** From these 45 published articles, participant level data were either publicly available or provided by authors for 890 participants aged 65 and over, with only 248 identified as aged 80 years and over. Authors of 30 publications to date generously contributed participant level data, whilst data from 15 publications were directly extracted from the published papers themselves. These data represent DLW studies conducted across all continents (with the exception of Antarctica) and in studies of ambulatory non-institutionalised people and those across a range of disease states. Overall, we have established that approximately 67% of the DLW data for the population of interest were unavailable.

**Table 3:** Details of participant-level data availability of studies of TEE using DLW in adults 65 years and over (total number of publications = 45)

Authors; year; country where study undertaken	Number of participant-level data (65 years and over)	Number of participant-level data (80 years and over)	Population studied
Prentice et al., 1989 [30]; England	14	7	Elderly mental health patients
Goran & Poehlman, 1992, 1992 [31, 32]; USA	10	0	Ambulatory non-institutionalised
Pullicino et al., 1993 [33]; England	1	0	Intravenously fed patients
Reilly et al., 1993 [34]; England	10	0	Ambulatory non-institutionalised
Kashiwazaki et al., 1995 [35]; Bolivia	2	0	Bolivian Aymara
Koea et al., 1995 [36]; New Zealand	2	0	Ambulatory patients and patients with sepsis
Pannemans et al., 1995 [37]; Netherlands	26	0	Ambulatory non-institutionalised
Rothenberg et al.; 2000,2002 [38, 39]; Sweden	21	21	Ambulatory non-institutionalised
Blanc et al., 2002, 2004 [13, 14]; Middleton et al., 2011 [19]; Manini et al., 2009, 2009 [17, 18]; Shahar et al., 2009, 2010 [20, 21]; Mackey et al., 2011 [16]; Cooper et al., 2013 [15]; USA	301	96	Ambulatory non-institutionalised
Rothenberg et al., 2003 [11]; Sweden	11	0	Ambulatory non-institutionalised
Slinde et al., 2003 [40]; Sweden	6	0	Chronic obstructive pulmonary disease patients
Hagfors et al.; 2005 [41]; Sweden	3	0	Rheumatoid arthritis patients
Arvidsson et al., 2006 [42] AND Slinde et al., 2006 [43]; Sweden	7	0	Chronic obstructive pulmonary disease patients
Frisard et al.; 2007 [12]; USA	146	100	Ambulatory non-institutionalised
Tooze et al., 2007 [44]; USA and Bradley et al., 2010 [45]; USA	65	0	Ambulatory non-institutionalised
Hertogh et al., 2008 [46]; Netherlands	16	0	Ambulatory non-institutionalised
Moshfegh et al., 2008 [47]; USA	48	0	Ambulatory non-institutionalised
Choquette et al.; 2009 [48]; Canada	9	0	Ambulatory non-institutionalised
Yamada et al., 2009, 2013 [49, 50]; Japan	30	8	Ambulatory non-institutionalised
Rothney et al., 2010 [51]; USA	2	0	Ambulatory non-institutionalised
Colbert et al., 2011, 2014 [52, 53]; USA	56	13	Ambulatory non-institutionalised
Ichihara et al., 2012 [54]; Japan	6	0	Advanced amyotrophic lateral sclerosis
Pontzer et al., 2012, 2015 [55, 56]; Tanzania	4	0	Hadza hunter-gatherers compared with Western population
Farooqi et al.; 2013, 2015, 2015 [57–59]; Sweden	13	2	Chronic obstructive pulmonary disease patients
Calabro et al.;2015 [60]; USA	22	0	Ambulatory non-institutionalised
Pfrimer et al., 2015 [61]; Brazil	30	0	Ambulatory non-institutionalised
Rollo et al.; 2015 [62]; Australia	4	0	Type 2 diabetes
Sridharan et al., 2016 [63]; England	13	1	Patients with chronic kidney disease
TOTAL	890	248	–

## Discussion

Our database search identified no reviews that had undertaken a systematic approach to identifying and collating the literature reporting TEE measured using DLW in an elderly population. Following the process of searching the literature, extracting data where reported, or contacting research teams of published studies, we identified 890 participant-level data points of TEE from individuals aged 65 years and over. For those over 80 years of age only 248 data points are available, with 100 of those contributed by Frisard et al. in the USA [12] and 96 from the Health, Ageing and Body Composition Study (Health ABC study) [13–21].

The search for participant-level data was complicated by a range of factors, including the changing composition of research teams originally undertaking the studies and authorship and dual publications from an original data set. During the time since the commencement of the DLW technique (35 years ago), there have been many changes in the research workforce and changes in technology. At the time of the original DLW studies, computers and electronic data storage were in their infancy. Subsequently email addresses were unavailable for many corresponding authors in the published record, and author contact details were unable to be traced through usual search engines. Additionally, data were not retrievable by the authors in some instances.

An additional challenge was the time delay associated with obtaining original data from research groups. Some authors were required to submit ethics applications to their institutions to enable data sharing for this research. Other groups were unable to search for data until team members returned from periods of leave, or until other projects were complete. As such, data collection for this review did not run to the timeframe expected.

We have identified that approximately 67% of DLW data sets are missing or unavailable. Although our review was limited to a defined age group, we expect that similar access issues would apply across other age groups, and animals where DLW studies have been undertaken. This is problematic since the costs associated with studies using DLW have been lost. Also, the findings of these studies would enable a more comprehensive and therefore representative data set to be established.

Current technology is now available internationally for data sharing, openly accessible data, and international data repositories which would mitigate the challenges of identifying and collating TEE data and the missed opportunities for its utilisation. With the cost at almost $AUD1000 per individual for isotopes alone to measure TEE using DLW, greater transparency in data reporting would support researchers internationally. Publication processes of some publishers require data access that support this ideal scenario. For example, the Public Library of Science (PLoS) have data reporting guidelines implemented whereby “all data and related metadata underlying the findings reported in a submitted manuscript should be deposited in an appropriate public repository, unless already provided as part of the submitted article” [22].

Alternatively, an international data repository should be considered. Data repositories and open access data have strengthened scientific research internationally over the past 20 years. Data sharing practices within the field of nutrition, and medicine more broadly are limited. Research data are not as available in repositories compared with other fields such as science and astronomy [23]. The Protein Data Bank is one example of an international collaboration for the deposition, processing and distribution of protein research. Founded in 1971, this repository stores three-dimensional structure data of biological macromolecules that have been determined under experimental conditions [24]. This repository captures and curates data using common practices and standards [24]. Although these data repositories are available at a specific project level, or sponsored from national bodies (National Research Infrastructure for Australia) [25], fewer are supported internationally.

Within healthcare, the Clinical Research Data Repository of the US National Institutes of Health has one of the largest publicly accessible research data repositories. Using a defined dictionary to unify terminologies from different data sources, this data repository includes in excess of 300 million rows of data [26]. Data retrieval can be made in identified form of an institutes’ own research participants, and the de-identified data of all participants [26]. This is an example of the potential for collaboration offered by repositories in the sciences. The largest accessible data set of DLW data that we identified was of the participants of the Women’s Health Institute study [27, 28] where data of 544 participants [27] and 450 participants [28] are stored. These data are available through an application process and engagement with a member of the research team.

The next step from the establishment of a data repository is data mining, “to use existing data to help build the evidence base for effective nutritional care” [29]. The collaboration of previous and future researchers to contribute to a similar repository for results of studies of human energy expenditure is needed. Such a database once established would enable these valuable data to be stored and analysed by researchers into the future.

This research had several key strengths including a wide-ranging database search, and search terms across the intervention and outcomes of interest, whilst not scoping for age. This increased the likelihood of identifying all relevant studies into our title/abstract review. The absence of date and language restrictions are further strengths as the data set has not been compromised by either of these commonly applied limitations.

### Recommendations for future research

Our planned analysis of the primary TEE data that we have obtained both from published sources and directly from authors will compare the accuracy of predictive equations commonly used in clinical practice against TEE determined through the DLW technique. This will be conducted for the broader elderly (65 years and over as we have scoped in this review) and for the older elderly (80 years and over) groups. As we have described, future research across the field of DLW would benefit from changes to process that ensure data are easily accessible. Data across clinical and other demographic subgroups could then be analysed to inform nutrition policy and practice at the population, community and individual patient/person level.

## Conclusion

Our review of the peer-reviewed literature identified studies that report TEE measured by DLW in individuals aged 65 years and over. Participant-level data points for further analysis have been obtained from published data and author contact for 890 individuals. However, the majority of original data were irretrievable due to inability to contact authors, or due to changes in technology. The development of an international data repository would facilitate greater access for researchers to integrate current research with previous findings, thus ensuring that TEE data obtained using high cost methods are not lost to future generations.

## Additional file

Additional file 1: PRISMA 2009 checklist. (DOC 63 kb)
